# A Retrospective Study on Antinuclear Antibody Patterns in Systemic Lupus Erythematosus Patients and Its Correlation With Serological Markers

**DOI:** 10.7759/cureus.50049

**Published:** 2023-12-06

**Authors:** Aishwarya Ramachandran, Kennedy Kumar, Rajeswari Sankaralingam, Saranya Chinnadurai, Balaji Chilukuri

**Affiliations:** 1 Microbiology, Sri Ramachandra Institute of Higher Education and Research, Chennai, IND; 2 Rheumatology, Sri Ramachandra Institute of Higher Education and Research, Chennai, IND

**Keywords:** anti-dsdna, rheumatic diseases, direct coomb's test, sle, indirect immunofluorescence, ana

## Abstract

Introduction: Systemic lupus erythematosus (SLE) is a multisystem autoimmune disease. Detection of antinuclear antibodies (ANAs) aids in the diagnosis of SLE. The indirect immunofluorescence (IIF) assay is often used a routine screening test for the detection of ANA. The pathogenic role and significance of various patterns produced in IIF is yet to be explored.

Aim: This study aimed to detect ANA patterns generated by IIF and correlate these patterns with specific antibodies detected by line immunoassay. We also investigated the significance of each ANA pattern and its association with specific serological SLE markers, such as complement molecules, anti-dsDNA, antiphospholipid antibody, and C-reactive protein (CRP), along with associations with direct Coombs test (DCT).

Materials and methods: We conducted a retrospective study that included 204 patients newly diagnosed with SLE according to the European Alliance of Associations for Rheumatology/American College of Rheumatology (EULAR/ACR) criteria. The detection and pattern determination of ANA was performed by IIF using HEp-20-10. Furthermore, line immunoassay was performed, and the antibody profile of each sample was obtained. Other immunodiagnostic markers were analyzed, including C3, C4, anti-dsDNA, antiphospholipid antibodies (anti-cardiolipin antibodies, anti-beta-2-glycoprotein I, and lupus anticoagulant), CRP, and DCT.

Results: Of the 204 samples, the most frequent ANA pattern observed was nucleus speckled (52.9%), followed by nucleus homogenous (27.5%), mixed (13.7%), and cytoplasm speckled (5.9%). The nucleus homogenous pattern showed the most pathogenic immune profile due to its close association with markers of disease activity, namely, high anti-dsDNA titer, low C3 level, and DCT positivity.

Conclusion: This study showed that the most common pattern associated with SLE is nucleus speckled, followed by the nucleus homogenous pattern. Based on associations with specific serological markers, the nucleus homogenous pattern may be linked to a high disease activity in SLE.

## Introduction

Systemic lupus erythematosus (SLE) is a systemic chronic inflammatory autoimmune disorder that affects multiple organs. It predominantly affects women, with a female-to-male ratio of 6-12:1 and a peak incidence during the reproductive age span [1,2]. One of the challenging aspects of SLE is the wide spectrum of clinical manifestations due to multisystem involvement and the progressive nature of the disease. Another hallmark of SLE is its association with a wide array of immune/serological markers. The identification of any serological abnormality and associated features contributes to the diagnosis of SLE and helps monitor the prognosis of the disease. 

The European Alliance of Associations for Rheumatology/American College of Rheumatology (EULAR/ACR) classification criteria for SLE include identification of antinuclear antibodies (ANAs), antiphospholipid antibody (APLA), and low complements levels [3]. The clinical criteria are grouped into seven clinical domains (constitutional, hematological (leukopenia, haemolytic anaemia, and thrombocytopenia), mucocutaneous, musculoskeletal, serosal, renal, and neuropsychiatric). 

In accordance with the criteria, the detection of ANAs is a key component considered under the immunological domain, and therefore, a positive ANA test is required for a patient to be classified as having SLE [4]. ANAs are a group of autoantibodies to cell nuclear antigens (nucleic acid, protein, and their complexes) [5]. In SLE, autoantibodies are frequently targeted against intracellular antigens of the cell nucleus (double-stranded DNA (dsDNA), histones, and other extractable nuclear antigens (ENAs)) and cell cytoplasm (ribosomal P protein) [6]. The criteria define ANA positivity as the presence of ANAs by immunofluorescence assay at a titre of 1:80 or greater on HEp-2 cells or another solid-phase assay with equivalent performance [7].

This study was performed to detect the most common ANA patterns among ANA-positive SLE patients, and the patterns were compared with the associated antibodies and other specific immunologic markers.

## Materials and methods

A retrospective study was conducted at the Department of Rheumatology and Microbiology at Sri Ramachandra Medical College and Research Institute, Chennai, India, after obtaining approval from its Institutional Ethics Committee (approval number: IEC-NI/23/AUG/88/55). Data collected from January 2019 to May 2023 were used for this study. The inclusion criteria were as follows: consecutive samples of ANA-positive SLE patients newly diagnosed based on the EULAR/ACR criteria, belonging to all age groups.

The exclusion criteria were as follows: ANA-negative SLE patients, duplicate samples from the same patient, and patients with no ANA pattern result. 

Patient demographic data and laboratory investigation reports of ANA titres, C3, C4, anti-cardiolipin antibodies (aCL), beta2-glycoprotein I (β2GP1), lupus anticoagulant (LAC), C-reactive protein (CRP), and direct Coombs test (DCT) results were collected from the Hospital Information System and electronic files from the Medical Records Department.

ANA indirect immunofluorescence

ANA by indirect immunofluorescence (IIF) testing is usually processed using BIOCHIP titre plane slides coated with HEp-20-10-HEp-2 and tissue sections of monkey hepatocytes-primate liver (Euroimmun, Germany). Slides containing fixed cells (HEp-20-10) were incubated with patient serum diluted with phosphate buffer saline. The dilution range starts from 1:100, and positive samples were further diluted by a factor of 10, 1:1000. The test procedure was then carried out as per the kit insert. Fluorescence and pattern were visualized using a fluorescence microscope at x400 magnification, and based on the intensity of the positive control, the fluorescence intensity of the positive samples was graded. The absence of a pattern and fluorescence on IIF was reported as ANA-negative. The results of this assay for all the samples were collected from the Hospital Information System and electronic files.

Line immunoassay

Serum samples that were positive for ANA by IIF were further tested for the presence of specific antibodies by dot blot/line immunoassay (LIA). LIA nylon strips with purified and recombinant antigens blotted as discrete lines at pre-located spots on the strip were used for this assay. A diluted serum sample (1:101) was used for this assay, and the test procedure was carried out as per the manufacturer’s instructions. The results of the LIA-ANA-Profile-17S (Euroimmun), which had the following antinuclear antigens, i.e., U1-small nuclear RNP, Smith (Sm), dsDNA, nucleosomes, histones, ribosomal P protein, PCNA (proliferating cell nuclear antigen, Ro52, SS-A/Ro60,SS-B/La, CENP (centromere protein)-B, Jo-1,Scl70, PM-Scl, AMA(antimitochondrial antibody)-M2, Ku, and a control band, were used for this study. In the presence of any specific autoantibody in the serum, a dark blue color, in proportion to the amount of specific autoantibody, is usually produced on the corresponding discrete line on the LIA strip. 

In Table 1, information on the methodology and kits used for the immunological investigations are presented. The testing protocol was followed, and conditions were maintained according to the manufacturer’s instructions.

**Table 1:** Methodology and kits used for the immunological investigations

Assay	Method	Kit used
Line immunoassay	Dot blot assay	EUROLINE ANA Profile 3 and EUROLINE ANA Profile 23 (Euroimmun)
Anti-dsDNA	ELISA	Anti-ds DNA–NCX ELISA Kit (Euroimmun)
Anti-cardiolipin antibodies - IgG and IgM	ELISA	Anti-cardiolipin ELISA (IgG and IgM) (Euroimmun)
Anti-beta2 glycoprotein I - IgG and IgM	ELISA	Anti-beta2-glycoprotein IgG and IgM (DiametraSrl)
Lupus anticoagulant	Dilute Russell Viper venom time assay	Lupus anticoagulant with LA1 screening/LA2 confirmation reagent (Siemens Diagnostics)
Complements (C3 and C4)	Immunoturbidimetry	AU analyser (Beckman Coulter)
C-reactive protein	Nephelometry	IMMAGE® Immunochemistry Systems (Beckman Coulter)
Direct Coombs test	Agglutination	Coombs Gel Card (Bio-Rad Laboratories)

The reference ranges were as follows: C3: 90-180 mg/dl, C4: 10-40 mg/dl, CRP: 0.0-0.8 mg/dl, anti-dsDNA antibody: <100 IU/ml, anti-cardiolipin antibody: IgG and IgM, <12 PL-IgG U/ml and <12 PL-IgM U/ml, respectively, and anti-beta-2 glycoprotein I IgG and IgM: <6 AU/ml.

All the immunodiagnostic assays mentioned in Table 1 were performed using suitable manufacturer-recommended positive and negative controls. The results were validated only when controls were satisfactory. The results of these assays and their association with ANA patterns were statistically analyzed using the chi-square test and ANOVA (analysis of variance).

## Results

A total of 204 SLE patients with positive IIF assay results were included in this study. Out of the 204 patients, 190 (93.1%) were women, and 14 (6.9%) were men, with ages ranging between 11 and 70 years. The mean age was 32 ± 13.19 years, and the median age was 30 (interquartile range, 15.4) years.

The demographic characteristics of the SLE patients included in this study are shown in Table 2.

**Table 2:** Demographic characteristics of the investigated SLE patients The demographic data of the investigated SLE patients are represented as n(%).

Age group, years	Female, n (%)	Male, n (%)	Total, n (%)
11- 20	22 (11.6)	8 (57.1)	30 (14.7)
21-30	74 (38.9)	4 (28.6)	78 (38.2)
31-40	52 (27.4)	0	52 (25.5)
41-50	28 (14.7)	0	28 (13.7)
51-60	12 (6.3)	2 (14.3)	14 (6.9)
60-70	2 (1.1)	0	2 (1)
Total	190 (93.1)	14 (6.9)	204 (100)

**Figure 1:**
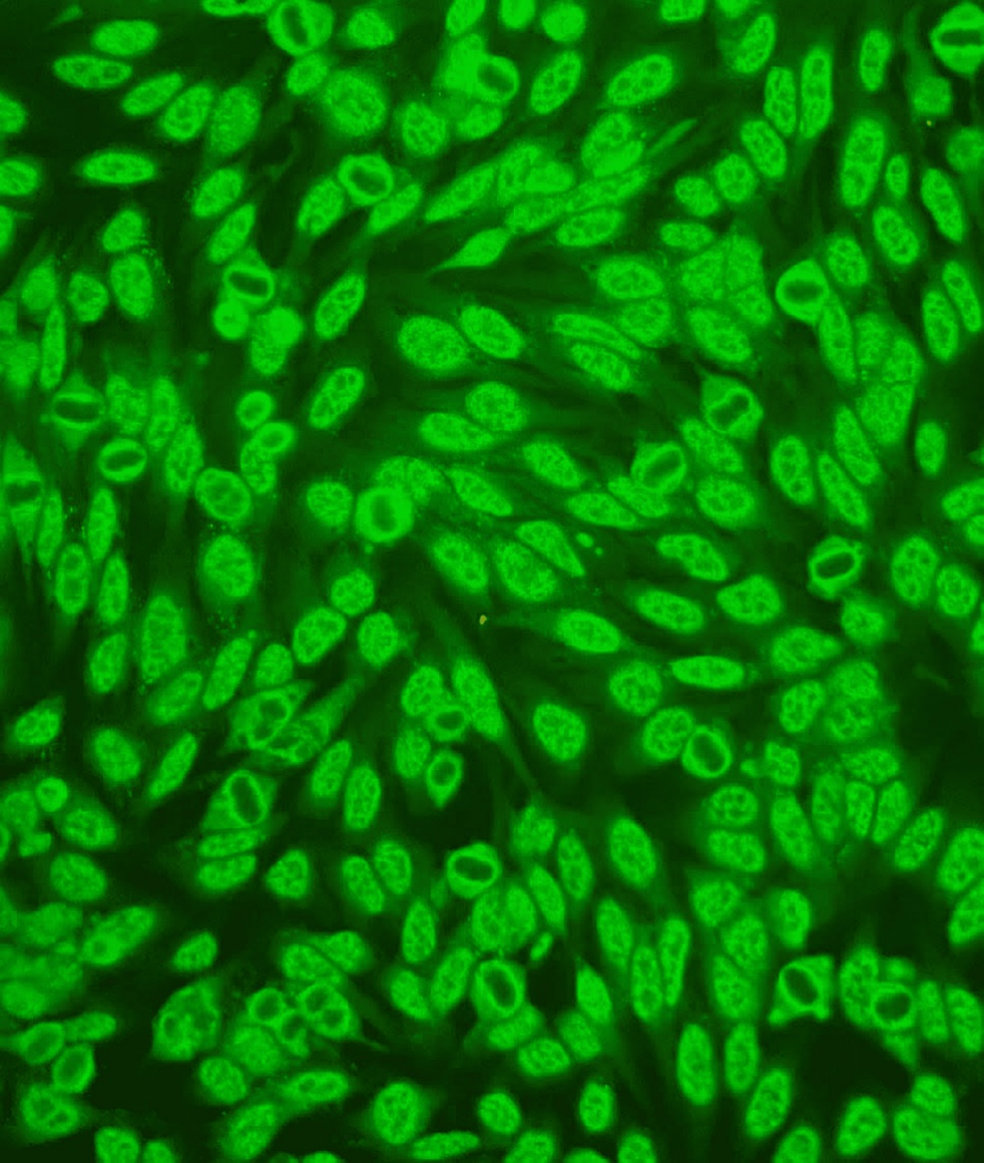
Immunofluorescence micrograph depicting the nucleus speckled pattern (×400) In this immunofluorescence micrograph, HEp-2 interphase cells show a coarse speckled pattern.

**Figure 2:**
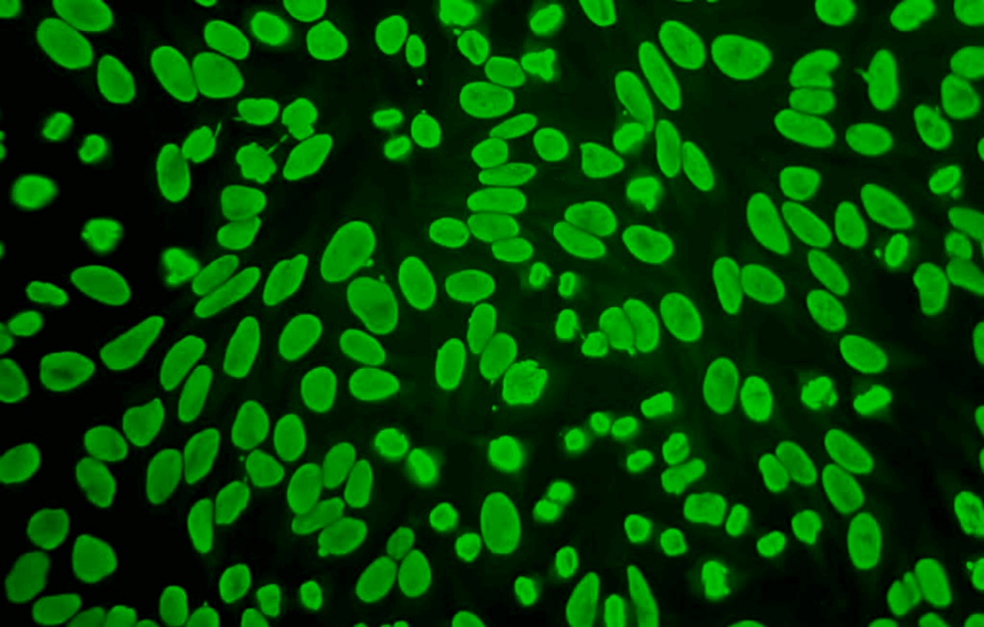
Immunofluorescence micrograph showing the nucleus homogenous pattern (×400) In this immunofluorescence micrograph, HEp-2 interphase cells show a hemogenous fluorescence of the cell nuclei. Mitotic cells are positive for condensed chromosomes.

**Figure 3:**
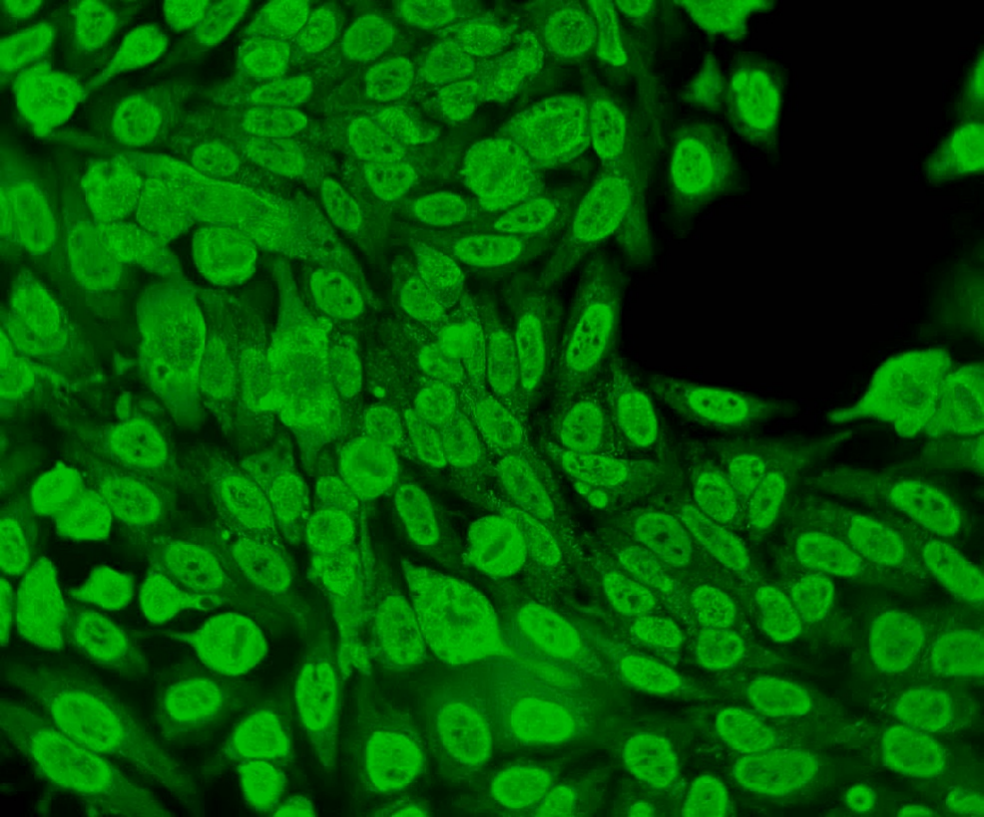
Immunofluorescence micrograph of the mixed pattern (×400) In this immunofluorescence micrograph, HEp-2 interphase cells show a nucleus speckled pattern along with fine speckled staining of the cytoplasm.

**Figure 4:**
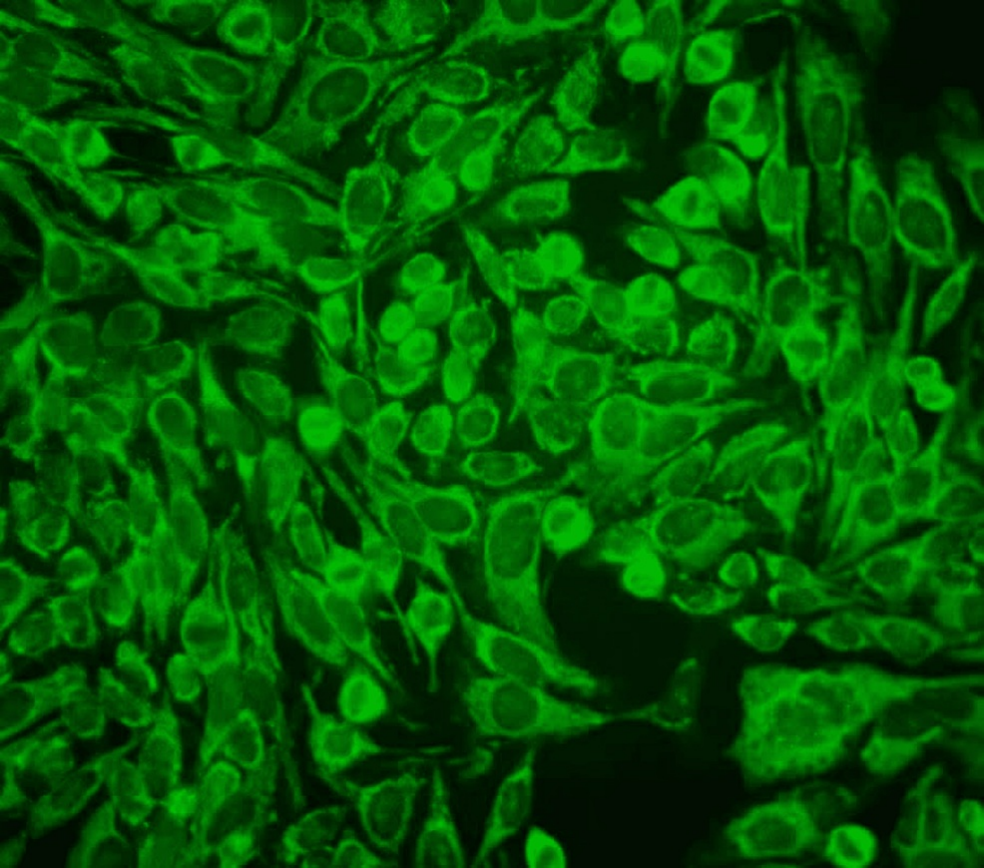
Immunofluorescence micrograph of the cytoplasm speckled pattern (×400) In this immunofluorescence micrograph, HEp-2 interphase cells show a smooth to fine speckled staining of the cytoplasm.

The ANA immunofluoresence microscopy staining patterns are depicted in Figures 1-4, and their distribution is presented in Table 3.

**Table 3:** Distribution of the observed ANA patterns (N=204) The distribution of observed antinuclear antibody (ANA) patterns among the investigated SLE are represented as n(%).

ANA pattern	n (%)
Nucleus speckled	108(52.9)
Nucleus homogeneous	56 (27.5)
Mixed – nucleus speckled and cytoplasm speckled	28 (13.7)
Cytoplasm speckled	12 (5.9)

The demographic association of each ANA pattern reported among the 190 females and 14 males is presented in Table 4.

**Table 4:** Demographic breakdown and association with ANA patterns The demographic association of ANA patterns are represented as n(%).

Age group, years	Pattern, n (%)								Total, n (%)
	Nucleus speckled		Nucleus homogenous		Mixed		Cytoplasm speckled		
										Female	Male	Female	Male	Female	Male	Female	Male
	11-20	12 (11.8)	4 (66.7)	2 (4)	4 (66.7)	6 (23.1)	0	2 (16.7)		0	30 (14.7)
21-30	38 (37.3)	2 (14.3)	24 (48)	2 (14.2)	6 (23.1)	0	6 (50)	0	78 (38.2)
31-40	30 (29.4)	0	14 (28)	0	6 (23.1)	0	2 (16.7)	0	52 (25.5)
41-50	18 (17.6)	0	4 (8)	0	6 (23.1)	0	0	0	28 (13.7)
51-60	4 (3.9)	0	4 (8)	0	2 (7.6)	2 (100)	2 (16.7)	0	14 (6.9)
60-70	0	0	2 (4)	0	0	0	0	0	2 (1)
Total	102 (53.7)	6 (42.8)	50 (26.3)	6 (42.8)	26 (13.7)	2 (14.4)	12 (5.9)	0	204 (100)

ANA patterns and antibody profile

**Figure 5:**
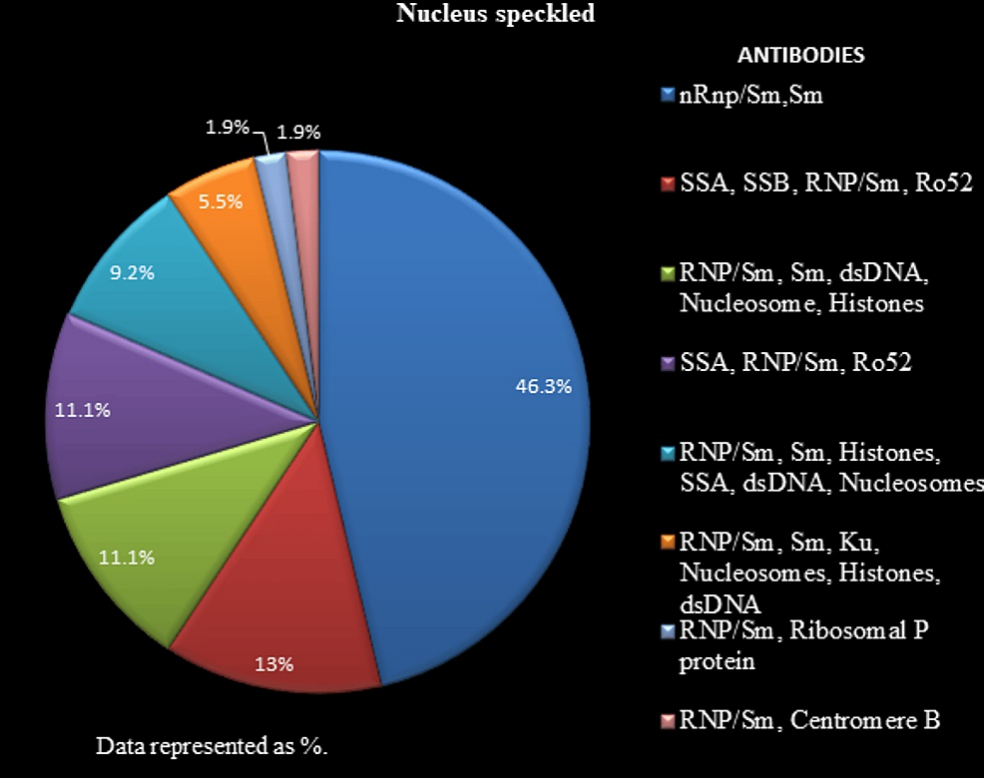
Antibody profile associated with the nucleus speckled pattern The antibodies associated with the nucleus speckled pattern are represented as %.

**Figure 6:**
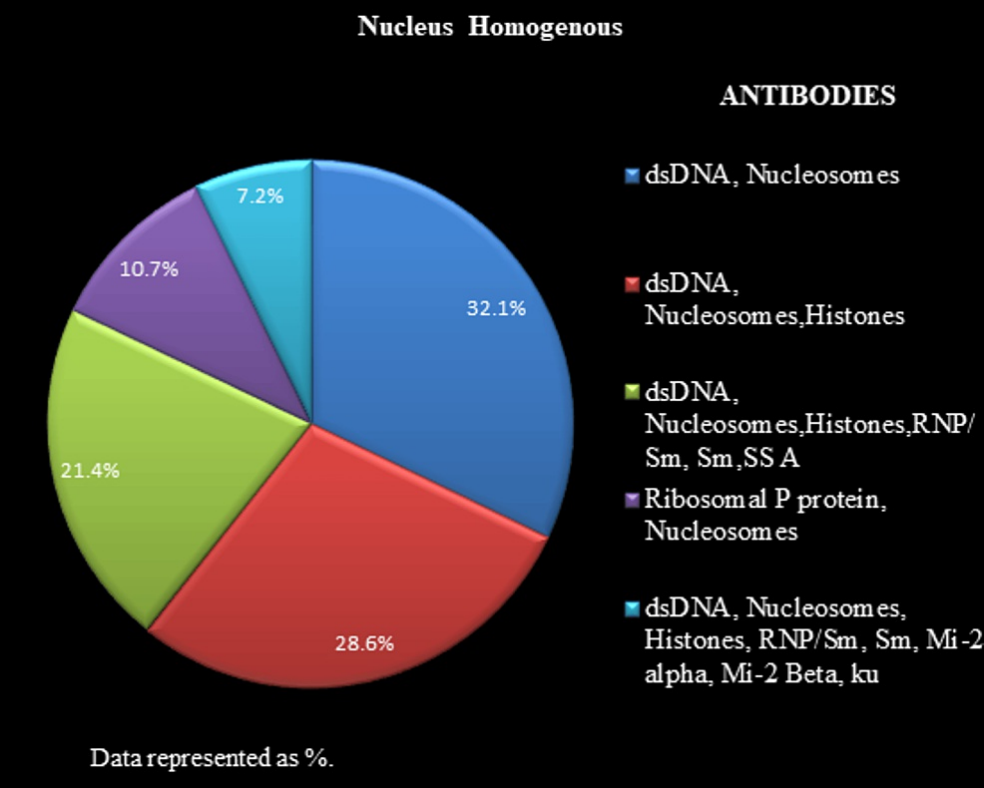
Antibody profile associated with a nucleus homogenous pattern The antibodies associated with the nucleus homogenous pattern are represented as %.

**Figure 7:**
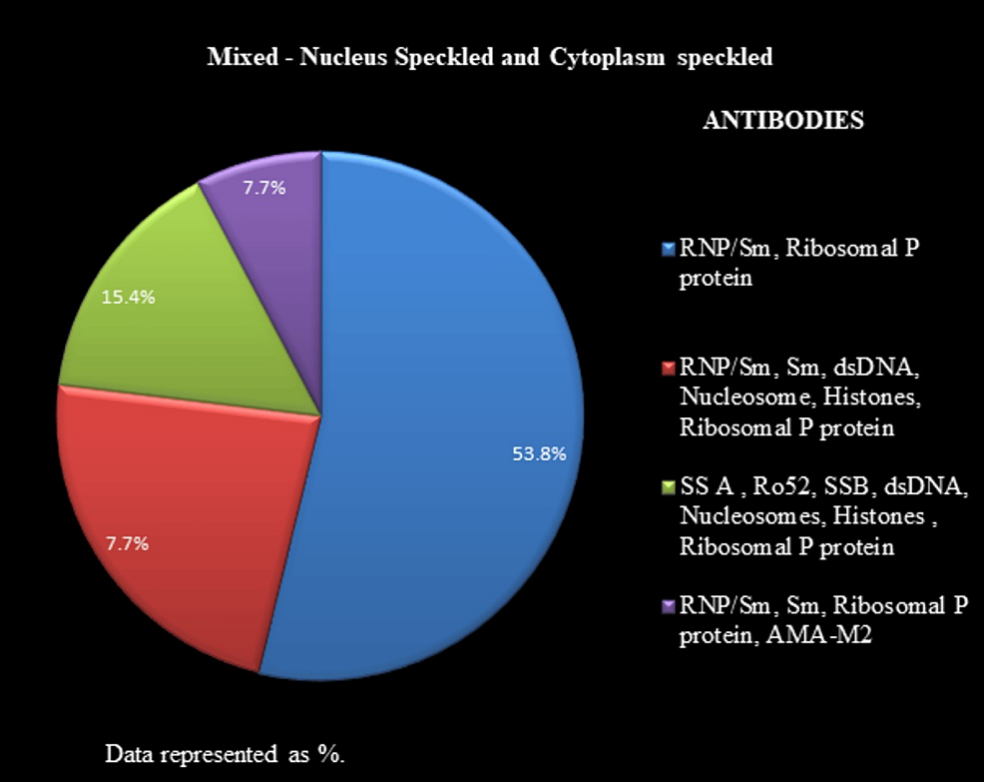
Antibody profile associated with the mixed pattern The antibodies associated with the mixed pattern, i.e., nucleus speckled and cytoplasm speckled, are represented as %.

All the 204 positive ANA IIF samples were further analyzed for the identification of specific antibodies by LIA. Figure 5 depicts the antibody proportions for nucleus speckled pattern samples. Figure 6 illustrates the antibody proportions for the nucleus homogenous pattern samples. Figure 7 shows the antibody proportions for the mixed pattern, i.e., nucleus speckled and cytoplasm speckled samples. ANA IIF, reported as cytoplasm speckled in 12 (5.9%) cases, showed anti-ribosomal P protein positivity in all instances, along with anti-SSA/ anti-Ro 52/anti-dsDNA positivity in four (1.96%) cases. 

The LIA findings are presented in Figures 5-7, listed with antibodies in a descending order of intensity.

Specific immunological markers

Table 5 summarizes the results of the analyses conducted to determine the presence of specific immunological markers in the SLE patients and their associations with ANA patterns.

**Table 5:** Association between ANA patterns and specific serological markers Data on CRP, aCL IgG and IgM, b2-IgG&IgM, lupus anticoagulant, and direct Coombs test are represented as n(%). The p-values for CRP, aCL IgG and IgM, b2-IgG&IgM, lupus anticoagulant, and direct Coombs test were calculated using the chi-square test.  A p-value <0.05 was considered statistically significant. Data on anti-dsDNA are represented as mean and standard deviation (SD). Data on C3 and C4 are represented as median and interquartile range (IQR). The p-values for anti-dsDNA, C3, and C4 were calculated using analysis of variance (ANOVA). A p-value <0.05 was considered statistically significant. ANA: antinuclear antibody, CRP: C-reactive protein, anti-dsDNA: anti–double stranded DNA, ACL: anti-cardiolipin antibody, IgM: immunoglobulin M, IgG: immunoglobulin G

Assay	Factor/level	Pattern				p-value
		Nucleus speckled (n=108)	Nucleus homogeneous (n=56)	Mixed (n=28)	Cytoplasm granular (n=12)	
CRP, n (%)	Positive	22 (20.4)	10 (17.9)	2(7.1)	4(33.3)	0.2
	Negative	82(76)	26 (46.4)	14(50)	4(33.3)	
	Not sent	4 (3.6)	30 (35.7)	12 (42.9)	4 (33.3)	
anti-dsDNA, IU/ml	Mean	300.2	473.85	357.14	627.8	<0.05
	SD	299.10	316.90	290.80	18.18	
C3, mg/dl	Median	57	70.8	76.8	117.2	<0.05
	IQR	65	63.75	60	45.75	
C4, mg/dl	Median	17	12.7	10	30.75	<0.05
	IQR	13.8	13.525	10.5	14.2	
ACL IgG, n (%)	Negative	56(51.8)	34(60.7)	20 (71.5)	4(33.3)	<0.05
	Positive	14 (13)	0	2 (7.1)	0	
	Not sent	38 (35.2)	22 (39.3)	6 (21.4)	8 (66.7)	
ACL IgM, n (%)	Negative	68(62.9)	32 (57.1)	20 (71.5)	4(33.3)	0.6
	Positive	2(1.9)	2 (3.6)	2 (7.1)	0	
	Not sent	38 (35.2)	22 (39.3)	6 (21.4)	8 (66.7)	
b2-IgG, n (%)	Negative	62 (57.4)	30 (53.6)	12 (42.9)	4(33.3)	0.6
	Positive	4 (3.7)	2 (3.6)	2 (7.1)	0	
	Not sent	12 (38.9)	21 (4.8)	14 (50)	8 (66.7)	
b2-IgM, n (%)	Negative	62 (55.2)	30 (53.6)	12 (42.9)	4(33.3)	0.6
	Positive	4 (1.9)	2 (3.6)	2 (7.1)	0	
	Not sent	12 (38.9)	24 (4.8)	14 (50)	8 (66.7)	
Lupus anticoagulant, n (%)	Negative	62 (55.2)	30 (53.6)	12 (42.9)	4(33.3)	0.6
	Positive	4 (1.9)	2 (3.6)	2 (7.1)	0	
	Not sent	12 (38.9)	24 (4.8)	14 (50)	8 (66.7)	
Direct Coombs test, n (%)	Negative	22(20.4)	4 (7.2)	4 (14.3)	4(33.3)	<0.05
	Positive	44 (40.7)	34 (60.7)	16 (57.1)	4(33.3)	
	Not sent	42(38.9)	18 (32.1)	8 (28.6)	4(33.3)	

## Discussion

This retrospective study explored the presence and significance of ANA in patients with SLE. Females were predominantly affected, with the proportion of females affected by SLE being notably high; the female-to-male ratio was 9:1. The study conducted on the epidemiology and sociodemographics of SLE reported that the prevalence of SLE was six times higher among women [8].

The age group most frequently diagnosed with SLE was the 21- to 30-year group, with 38.2% (n=78) of patients falling into this age bracket. A cross-sectional study conducted in in a tertiary care hospital in North India indicated that the peak incidence of SLE occurred in the 21- to 30-year age group [9]. 

ANA-IIF is a widely used screening assay for ANA detection [10,11]. In our study of 204 SLE patients, we first identified and reported the ANA pattern by IIF. The predominant ANA patterns were nucleus speckled (n=108, 52.9%) and nucleus homogenous (n=56, 27.5%), followed by mixed (nucleus speckled and cytoplasm speckled) (n=28, 13.7%) and cytoplasm speckled (n=12, 5.9%) patterns.

Aligning with our findings, a research in Saudi Arabia found nucleus speckled pattern (79.5%) as the most frequent, followed by the homogeneous (11.4%) pattern [12]. Conversely, a Swedish study with 219 patients recorded the frequency of the nucleus speckled pattern at only 22%; the nucleus homogeneous pattern (54.3%) was more prevalent than the speckled pattern, with the mixed (homogeneous and speckled) pattern coming in third at 11.0% [13].Similarly, in a study conducted in South India, majority of SLE patients showed a nucleus homogenous pattern (31%), followed by a nucleus speckled pattern (17%)[14]. Data from the Systemic Lupus International Collaborating Clinics, detailing 1,137 newly diagnosed SLE patients, showed that the nucleus speckled pattern constituted less than 1% of the total, while the majority exhibited other nuclear (77.1%) or a combination of cytoplasmic and mitotic cell (15.1%) patterns [15]. This variation in ANA patterns in SLE may stem from genetic differences and clinical disparities, which could be influenced by disease activity levels and concurrent steroid medications [16,17,18].

LIA is used to identify specific antibodies and carry out autoantibody profiling. It is recognized for high sensitivity and specificity [19,20]. For the nucleus speckled pattern (n=108,52.9%), the most prevalent antinuclear autoreactivity was directed toward nRnp/Sm, Sm (51%) and SSA, SSB, RNP/Sm, and Ro52 (14.7%), among others. Every case with a nucleus speckled pattern exhibited an association with antibodies against RNP/Sm, albeit with varying intensities.

For samples indicating a nucleus homogenous pattern (n=56, 27.5%) pattern, anti-dsDNA and nucleosomes were the most commonly identified antibodies, present in 92.8% of the cases in various combinations and intensities. Studies have reported the presence of antibodies against dsDNA, nucleosomes, and histones in 75% of cases with a nucleus homogenous pattern [21].

The mixed (nuclear speckled and cytoplasm speckled) pattern samples (n= 28, 13.7%) primarily showed the presence of antibodies, such as RNP/Sm and ribosomal P protein, among others, based on the LIA findings. 

Nuclear speckled and cytoplasm speckled patterns are treated as distinct entities and not combined under the International Consensus on ANA patterns [22]. This pattern was associated with both nuclear and cyotplasmic antibodies: RNP/Sm, anti-ribosomal P protein at 53.8%.

Abnormal levels of C3 and C4 in association with SLE result from the activation of a complement system by autoantibodies. The measurement of serum C3 and C4 levels has been used to assess SLE disease activity using the Systemic Lupus Erythematosus Disease Activity Index (SLEDAI) [23]. In this study, when comparing the levels of C3 and C4 with ANA patterns, both C3 and C4 levels were significantly lower in patients with a nuclear pattern (speckled and homogenous (p < 0.05) and mixed pattern (p < 0.05), respectively). Analyzing C3 and C4 levels in SLE patients with nucleus speckled and mixed patterns provides a reliable marker for monitoring disease status.

Anti-dsDNA is recognized as a highly specific marker for SLE, and anti-dsDNA titers are useful to determine the extent of organ involvement and disease activity [24]. Significantly elevated anti-dsDNA titers were associated with the cytoplasm speckled pattern and nucleus homogenous pattern (p < 0.05). The cytoplasm speckled pattern finding might be less significant due to the small sample size in this study; however, this pattern is exclusive to females, and all these samples tested positive for anti-ribosomal P protein and high titers of anti-dsDNA.

Among the nucleus homogenous group, 80% of patients with raised CRP levels also displayed elevated anti-dsDNA titers, averaging 598.5. CRP levels in SLE typically correspond with disease activity, and thus, studies suggest that increased anti-dsDNA antibodies and high CRP levels occur in SLE with increased disease activity [25]. The nucleus homogenous pattern might serve as a marker of high disease activity.

In our study, APLA positivity accounted for 13.7% (n=28) of the total sample size. Contrasting with our findings, other studies have reported the prevalence of APLA in SLE to be 24-50% [26,27]. The aCL IgG antibodies were present in 13%(n=14) of patients with the nucleus speckled pattern, 7.1% (n=2) of patients with a mixed pattern, and 0% of both nucleus homogenous pattern and cytoplasm speckled pattern (p < 0.05). IgM APLA was less frequent than IgG. The presence of APLA (aCL, β2GPI, and LAC) in SLE is usually associated with an increased risk of thrombotic events [28]. Among the samples positive for APLA, 78.6% (n=22) were DCT-positive.

The DCT detects antibodies adhering to red blood cell surfaces. The test is positive in autoimmune hemolytic anemia and occasionally even when haemolysis is absent [29]. In 2012, the Systemic Lupus International Collaborating Clinics (SLICC) included a positive DCT, in the absence of hemolytic anaemia, as a criterion for diagnosing SLE. The DCT was positive in 60.7% (n=34) of patients with the nucleus homogenous pattern, 57.1% (n=16) of patients with the mixed pattern, and 40.7%(n=44) of patients with the nucleus speckled pattern (p < 0.05). Among samples displaying a nucleus homogenous pattern with increased anti-dsDNA titers, a positive DCT was observed in 88.2% (n=30) of cases, compared with 34.1% (n=22) and 25% (n=10) in the nucleus speckled and mixed groups, respectively. The presence of anti-dsDNA might be associated with a positive DCT, especially among patients with a nucleus homogenous pattern.

Limitation

The small sample size for the cytoplasm speckled group might influence the validity of the findings related to that particular pattern.

## Conclusions

Screening for ANA by IIF can serve as a primary test in the diagnosis of SLE. The most common age of presentation was between 21 and 30 years, with the nucleus speckled pattern being the most observed among SLE patients, followed by the nucleus homogenous pattern. This research underscores the value of conducting specific serological assays and understanding their associations with different ANA patterns. A nucleus homogenous pattern may be associated with a high anti-dsDNA titer, a larger number of samples with a positive DCT, and elevated CRP, indicating increased disease activity in these patients.
